# Real-world challenges associated with the use of four common systemic glucocorticoids in a United States IgAN cohort

**DOI:** 10.3389/fneph.2025.1574239

**Published:** 2025-04-24

**Authors:** Giancarlo Pesce, Mit Patel, Gaelle Gusto, Ananth Kadambi, Aastha Chandak, Terri Madison

**Affiliations:** ^1^ Real-World Evidence & Modeling Solutions, Certara, Milan, Italy; ^2^ U.S. Health Economics & Outcomes Research, Calliditas Therapeutics, New York, NY, United States; ^3^ Real-World Evidence & Modeling Solutions, Certara, Paris, France; ^4^ Real-World Evidence & Modeling Solutions, Certara, New York, NY, United States

**Keywords:** IgAN, glucocorticoids, healthcare resource utilization, kidney function, glomerulopathy

## Abstract

**Objectives:**

To understand the difference in adverse events (AEs), healthcare resource utilization (HCRU), and kidney failure rates in immunoglobulin A nephropathy (IgAN) patients who initiated systemic glucocorticoid (SGC) treatment compared with those who did not.

**Methods:**

The overall cohort was selected from patients with IgAN (ICD-10 codes N02.8 and N04.1) identified in the TriNetX Dataworks database between January 2011 and May 2022. New initiators of dexamethasone, prednisone, prednisolone, or methylprednisolone (SGC cohort) were propensity score (PS) matched 1:1 with patients who did not receive SGC (non-SGC cohort) based on their characteristics at diagnosis. The index date was the date of SGC initiation; for the non-SGC cohort, a pseudo-index date was assigned using the same lag from diagnosis to index date as their PS-matched pairs. Patients with kidney failure before the index/pseudo-index date and their 1:1 PS-matched pairs were excluded.

**Results:**

The final analysis was conducted in 802 patients (401 PS-matched pairs, mean age 41.2 years, 55% male). Median duration of follow-up was 3.5 and 3.1 years for the SGC and non-SGC cohorts, respectively. Compared with the non-SGC cohort, patients in the SGC cohort had greater frequency of several AEs, including severe infections, greater annualized HCRU and costs, and greater incidence of kidney failure.

**Conclusions:**

This study found that SGC therapy may increase adverse reactions and HCRU in IgAN patients, while comparatively providing no beneficial effects on preserving kidney function.

## Highlights

- To date there have been a few randomized clinical trials (RCTs) to evaluate the safety and efficacy of SGC added to optimized best supportive care in IgAN patients at risk disease progression. While these RCTs consistently demonstrated the safety impact of SGC, they report contradictory evidence on efficacy and long-term preservation of kidney function.
- Our study demonstrated that IgAN patients treated with four common SGC (dexamethasone, prednisone, prednisolone, or methylprednisolone) have higher proportion of adverse events, including severe infections requiring hospitalizations, and HCRU compared with patients not treated with SGC, with comparatively no benefit in preserving eGFR.
- The study results indicate that, given the limited evidence on long-term effectiveness of SGC, treatment toxicity should be carefully considered before exposing patients with IgAN to SGC.

## Introduction

Immunoglobulin A nephropathy (IgAN) is the most common primary glomerular disease. IgAN is an autoimmune condition that causes chronic kidney inflammation and a gradual deterioration of glomerular function that eventually progresses to kidney failure within 10 to 15 years (1–3). In the United States (US), the annual incidence of IgAN is 1.29 new cases per 100,000 (4), and over one-hundred-thousand people live with it (5). The complications of advanced chronic kidney disease (CKD) and kidney failure in IgAN patients include the requirement for dialysis or transplantation, poor health-related quality of life, and increased clinical burden and mortality (4–7).

Prior to IgAN-specific treatments approved since late 2021, management of IgAN has been primarily focused on supportive care to slow the rate of disease progression. The Kidney Disease: Improving Global Outcomes (KDIGO) guidelines recommend controlling blood pressure and using renin angiotensin system (RAS) blockade as the first management strategy for all IgAN patients with proteinuria >0.5 g/day (1). For patients at high risk of progressive CKD despite maximal supportive care, the 2021 guidelines recommend patients be offered participation in a clinical trial, and, if not feasible, suggest that patients can be considered for a 6-month course of systemic glucocorticoids (SGC). The KDIGO guidelines emphasize discussing the important risk of treatment-emergent toxicity with patients and considering contraindications, such as severe renal dysfunction or certain comorbidities, such as latent infections or severe osteoporosis, before SGC treatment initiation (1). The 2021 KDIGO guidelines also conclude that there are uncertainties regarding the safety and efficacy of current immunosuppressive treatment choices (1).

Two multicenter, randomized, controlled trials studied the safety and effectiveness of immunosuppressive therapy with SGC in patients with IgAN: the STOP-IgAN and the TESTING trials (8–10). The STOP-IgAN trial, published in 2015 and considered in 2021 KDIGO guidelines, showed that patients with IgAN treated with immunosuppressive therapy in addition to supportive care had a significant increase in severe infections as compared to patients treated with supportive care alone, and had no significant change in the rate of decrease in eGFR (9, 10). The TESTING trial, published in 2022, reported a greater preservation of kidney function in patients treated with methylprednisolone vs. placebo, but also a higher incidence of serious adverse events (AEs) (8).

Beyond clinical trials, there is limited information describing the real-world use of SGC for IgAN treatment. In this study, we examine the incidence of a targeted list of 20 AEs commonly associated with SGC, healthcare resource utilization (HCRU) rates and costs, and rates of kidney failure in a real-world cohort of US patients with IgAN, comparing new initiators of four of the most commonly used SGC – namely dexamethasone, prednisone, prednisolone, and methylprednisolone – with patients who have never initiated these four SGC.

## Methods

### Study design and population

This was a non-interventional, retrospective cohort study using the US TriNetX Dataworks (https://trinetx.com/), a research database that at the time this dataset was curated, contained de-identified electronic medical records (EMR) for over 94 million patients across four US census regions and 58 healthcare organizations (HCOs). Available data include patient demographics, procedures, medications, laboratory results, vital status, genomics, and oncology records. The average refresh of data from contributing HCOs and other providers is approximately once per month.

The cohort was selected from a total of 19,687 patients with incident IgAN. At the time this study was conducted, there was no specific ICD-10 code for IgAN. A targeted literature review conducted to identify recently published real-world studies in US-based IgAN patients provided limited insight into ICD-10 code selection, as only two publications were identified (5, 11), one of which used natural language processing [NLP] to identify IgAN. Since NLP is not possible in TriNetX Dataworks, we used a definition aligned with the results of a 2021 survey conducted on behalf of Calliditas by Spherix Insights, where the majority (78% of n=409) of US-based HCPs surveyed used the ICD-10 code N02.8 (recurrent and persistent hematuria with other morphologic changes) to identify IgAN [internal data on file], and supplemented with N04.1 (nephrotic syndrome, a code also observed to also be used for IgAN that was identified by the Spherix survey). Using these two proxy codes, we identified incident IgAN patients between January 1, 2011, and May 4, 2022. Eligible patients were required to have: no prior IgAN diagnosis before 2011, another diagnosis of IgAN with the same ICD-10 code within 30-180 days, at least 12 months of EMR history available prior to and after initial IgAN diagnosis, no kidney failure (i.e., diagnosis of CKD stage 5, or eGFR <15 mL/min/1.73 m^2^, or kidney transplant) prior to or on the initial date of IgAN diagnosis, and no treatment with systemic formulations of four SGCs (dexamethasone, prednisone, prednisolone, methylprednisolone) prior to the date of IgAN diagnosis (SGC-naïve).

### SGC exposure cohorts

Patients were then divided in SGC and non-SGC cohorts based on whether or not they received treatment with at least one of the four commonly used SGCs after the initial IgAN diagnosis. To promote balance between cohorts, new initiators of any SGC were matched with patients who never received SGC using a propensity score (PS) approach based on key characteristics at diagnosis, including age, sex, type of IgAN diagnosis (N02.8 vs N04.1), CKD stage, year of IgAN diagnosis (2011-2016, 2017-2018, 2019-2020, 2021-2022), hypertension, and Charlson Comorbidity Index (CCI) at baseline. The ICD-10 codes for the CCI components and other comorbidities of interest are provided in the "**Appendix**". A threshold of 10% in standardized mean difference was used to ascertain the balance of covariate distribution after PS matching. To avoid immortal-time bias, the index date in the SGC cohort was set as the time SGCs were initiated; for the non-SGC cohort, a pseudo-index date was assigned using the same lag from diagnosis to index date as their 1:1 PS-matched patient in the SGC cohort.

### Statistical analyses

Differences in the cumulative incidence of individual AEs were analyzed using the chi-square test or Fisher’s exact test. Time to kidney failure was analyzed using a Kaplan-Meier approach. Cox proportional hazard models were used to compare risk of kidney failure, adjusting for unbalanced variables at index/pseudo-index date.

Multivariable analyses were used to compare the incidence of kidney failure in PS-matched cohorts of patients exposed vs. not exposed to any of the four common SGCs. Comorbidities at the index/pseudo-index date that were not balanced in the PS-matched cohorts (i.e., CCI, hypertension, asthma, connective pulmonary disease, moderate or severe renal disease, cancer) were considered for further adjustment. The outcome of interest was post-index kidney failure, which was defined as diagnosis of end stage CKD via ICD-10 code, occurrence of transplant procedures, or eGFR measurements <15 ml/min/1.73m^2^. Kidney failure occurrence was assessed annually up to year five. A stepwise selection was conducted to retain the most significant variables amongst the candidates with a threshold of 0.3 to enter the model and of 0.35 to remain in the model (12). Significance was assumed at a p-value <0.05. For 1-5 years of follow-up, where the proportional hazards assumption held, a Fine and Gray model was implemented using death as the competing event to the outcome of interest and SGC as the exposure of interest (13). Results were expressed in adjusted hazard ratios (aHR, with 95% confidence intervals) for the Fine and Gray model.

The number of HCRU categories (hospitalizations, emergency room [ER] visits, and outpatient visits) recorded after the index/pseudo-index date were annualized. Differences in mean HCRU rates and costs were analyzed using the Kruskal-Wallis test. For each HCRU category considered, cost was estimated by multiplying the number of visits of the category of interest (or number of overnight stays for inpatient hospital cost) by the average cost of the category adjusted to 2022 USD using the medical care component of the consumer price index (14–16).

For all analyses, missing values were reported as missing with no replacement or imputation. Descriptive statistics for continuous variables included the available n, and descriptive statistics for categorical variables included a category of “missing” when applicable.

### Sensitivity analyses

The analyses were replicated in the strata of PS-matched IgAN patients who were identified using the ICD-10 diagnosis code N02.8 only (i.e., excluding patients identified through the N04.1 diagnosis code). Less than 10% of the study cohort (8.5% [SGC cohort] and 8.7% [non-SGC cohort]) were excluded in the sensitivity analyses.

## Results

### Study population

**Figure 1:**
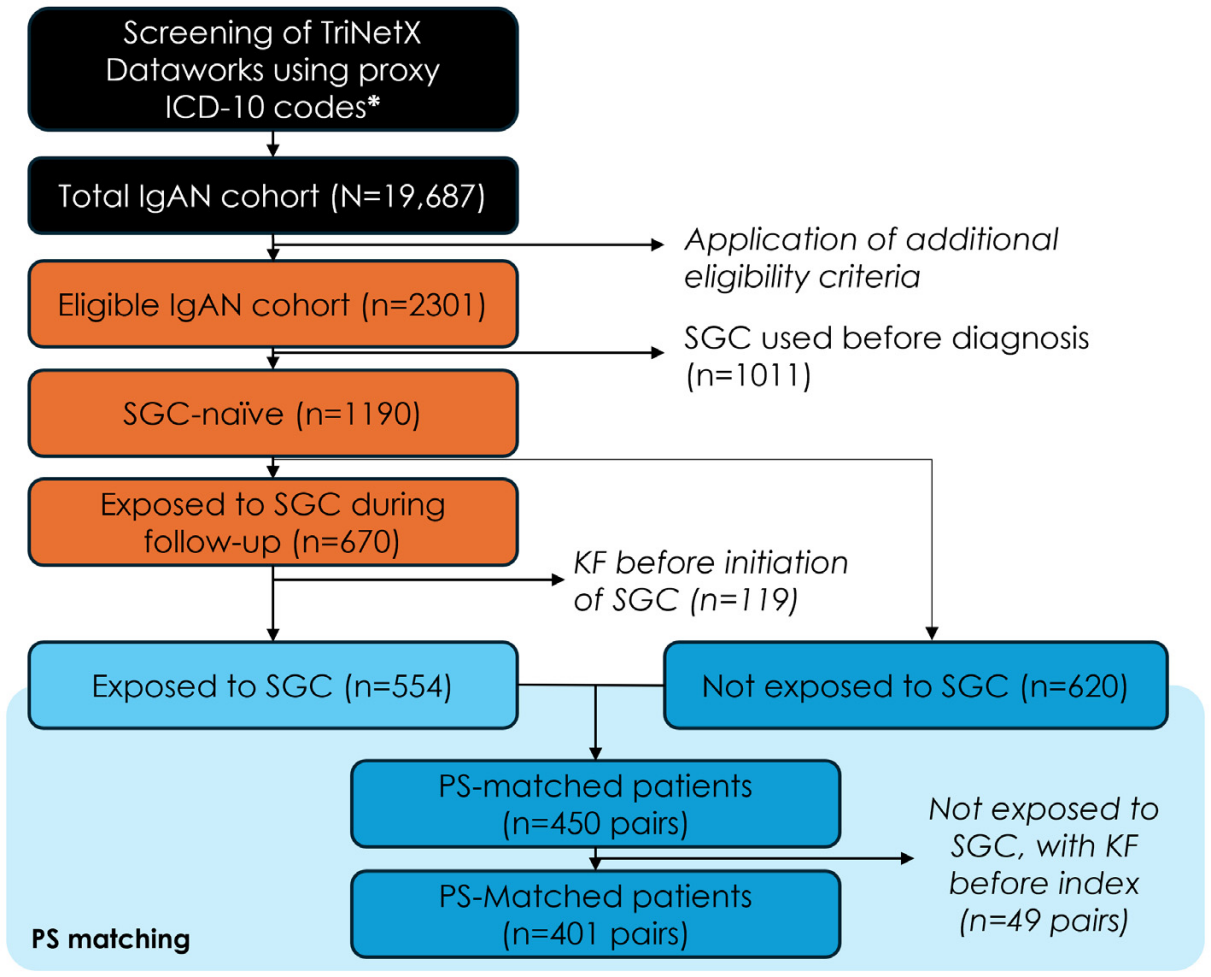
Study population flow-chart. ICD-10, international classification of diseases, 10^th^ revision; IgAN, immunoglobulin-A nephropathy; SGC, systemic glucocorticoids; KF, kidney failure; PS, propensity score. Additional eligibility criteria: first claim with diagnosis code N02.8 or N04.1 (=index date) between Jan 1, 2011 and May 4, 2022, n=19,687; confirmation diagnosis within 30 and 180 days from index date, n=5,827; 12-month of electronic medical record history available before index date, n=4,177; 12-month of electronic medical record history available after the index date (if the patient died or had kidney failure, they were followed until the date of kidney failure or death, whichever occurred last), n=3,956; without kidney failure before the index date, n=2,301.

Overall, 2,301 of patients initially selected based on the ICD-10 codes met the other study eligibility criteria, and 1,190 of these had not received any of the four common SGCs at diagnosis. Among SGC-naïve patients, n=670 initiated treatment with one of the four SGCs after IgAN diagnosis (SGC cohort), while n=620 were never treated with these four SGC after diagnosis (non-SGC cohort). SGC cohort patients who had kidney failure before the first SGC treatment (n=119) and all PS-matched pairs (n=49) for which the patients in the non-SGC cohort had kidney failure prior to the pseudo-index date were excluded from the analysis. After implementing the PS approach, the final analysis included 802 patients (401 PS-matched pairs) (**Figure 1**). The distribution of baseline characteristics at IgAN diagnosis in the SGC and non-SGC cohorts before and after PS matching is included in the **Supplementary Table S1**.

The median duration of follow-up after index/pseudo-index date was 3.5 and 3.1 years for the SGC and non-SGC cohorts, respectively. The SGC and non-SGC cohorts had a similar age (mean: 41.9 vs 40.8 years), proportion of female sex (45%), and CKD stage at index/pseudo-index (stage 3+: 33% vs 31%, **Table 1**). Patients initiating SGC treatment, however, had on average more comorbidities (mean CCI: 1.95 vs 1.56), and a greater proportion of hypertension (62% vs 54%), asthma (8% vs 4%), chronic obstructive pulmonary disease (COPD) (12% vs 6%), moderate/severe renal disease (52% vs 45%), and cancer (5% vs 2%) at index date compared with patients who did not initiate SGC.

**Table 1:** Characteristics at index of propensity score matched cohorts.

Baseline characteristics	SGC (n=401)	Non-SGC (n=401)	*p*-value
**Age, mean (SD)**	41.9 (18.5)	40.8 (17.8)	0.37
**Sex (females), n (%)**	181 (45.1)	182 (45.4)	0.94
**Patient Regional Location**			0.30
Midwest	90 (22.4)	70 (17.5)	
Northeast	144 (35.9)	142 (35.4)	
South	110 (27.4)	127 (31.7)	
West	57 (14.2)	61 (15.2)	
Unknown			
**Race, n (%)**			0.69
American Indian or Alaska Native	2 (0.5)	1 (0.3)	
Asian	50 (12.5)	42 (10.5)	
Black or African American	34 (8.5)	31 (7.7)	
White	242 (60.4)	261 (65.1)	
Unknown/Unspecified	73 (18.2)	66 (16.5)	
**Hispanic/Latino, n (%)**	58 (14.5%)	55 (13.7)	0.95
**CKD stage, n (%)**			0.35
1-2	123 (30.7)	132 (32.9)	
3	93 (23.2)	96 (23.9)	
4	41 (10.2)	27 (6.7)	
Missing	144 (35.9)	146 (36.4)	
**eGFR, mean (SD)**	64.6 (32.3)	69.9 (30.1)	0.06
**CCI**			**0.002**
Mean (SD)	1.95 (2.1)	1.56 (1.9)	
**CCI category, n (%)**			**0.02**
0	129 (32.2)	171 (42.6)	
1	36 (9.0)	32 (8.0)	
2	136 (33.9)	123 (30.7)	
3+	100 (24.9)	75 (18.7)	
Comorbidities, n (%)			
Hypertension	247 (61.6)	216 (53.9)	**0.03**
Asthma	33 (8.2)	17 (4.2)	**0.02**
Congestive heart failure	17 (4.2)	14 (3.5)	0.58
Peripheral vascular disease	17 (4.2)	18 (4.5)	0.86
Cerebrovascular disease	13 (3.2)	6 (1.5)	0.10
Chronic pulmonary disease	48 (12.0)	25 (6.2)	**0.005**
Connective tissue disease	15 (3.7)	14 (3.5)	0.85
Mild liver disease	31 (7.7)	29 (7.2)	0.79
Diabetes without complication	39 (9.7)	46 (11.5)	0.42
Moderate or severe renal disease	209 (52.1)	179 (44.6)	**0.03**
Diabetes with complication	23 (5.7)	20 (5.0)	0.64
Cancer	21 (5.2)	9 (2.2)	**0.03**
Moderate or severe liver disease	13 (3.2)	5 (1.3)	0.06
**RAS inhibitor therapy^**	126 (31.4)	121 (30.2)	0.70

CCI, Charlson Comorbidity Index; CKD, chronic kidney disease; eGFR: estimated glomerular filtration ratio; RAS, renin angiotensin system; SD, standard deviation; SGC, systemic glucocorticoids. ^in the 12 months prior of the index date.

Statistically significant p-values (p < 0.05) are indicated in bold.

### Adverse events

Sixteen of the 20 AEs assessed occurred in at least ten of the 802 patients. Patients who received SGC experienced a significantly increased incidence of all AEs except for fractures, new onset of diabetes, and reported onset of glaucoma (**Table 2**).

**Table 2:** Adverse events in IgAN patients by use of systemic glucocorticoids.

AEs, n (%)	SGC (n=401)	Non-SGC (n=401)	*p*-value
**Hypertension**	294 (73.3)	252 (62.8)	**0.002**
Incident hypertension*	65 (42.2)	53 (28.7)	**0.009**
**Arthralgia**	131 (32.7)	83 (20.7)	**<0.001**
**Dyspnea**	109 (27.2)	46 (11.5)	**<0.001**
**Fatigue**	99 (24.7)	57 (14.2)	**<0.001**
**URTI**	87 (21.7)	50 (12.5)	**0.001**
**New onset of diabetes mellitus**	67 (16.7)	57 (14.2)	0.330
**Peripheral/face edema**	59 (14.7)	17 (4.2)	**<0.001**
**Increase in WBC count**	36 (9.0)	13 (3.2)	**0.001**
**Dermatitis**	35 (8.7)	21 (5.2)	**0.050**
**Dyspepsia**	31 (7.7)	15 (3.7)	**0.020**
**Confirmed fracture**	21 (5.2)	24 (6.0)	0.650
**Acne**	19 (4.7)	8 (2.0)	**0.030**
**Weight increased**	17 (4.2)	5 (1.3)	**0.010**
**Severe infection requiring hospitalization**	14 (3.5)	1 (0.3)	**0.001**
**GI bleeding requiring hospitalization**	9 (2.2)	2 (0.5)	**0.030**
**Reported onset of glaucoma**	8 (2.0)	10 (2.5)	0.630

AEs ordered from high to low according to SGC treatment group. Frequency of four AEs that did not occur in at least 10 patients (i.e., dementia n=3, paraplegia/hemiplegia n=2, acquired immunodeficiency syndrome n=4, metastatic solid tumor, n=2) were not reported.

*Among patients without hypertension diagnosis before index/pseudo-index date.

AE, adverse event; GI, gastrointestinal; SGC, systemic glucocorticoids; URTI, upper respiratory tract infection; WBC, white blood cell.

Statistically significant p-values (p < 0.05) are indicated in bold.

### Healthcare resource utilization and costs

**Figure 2:**
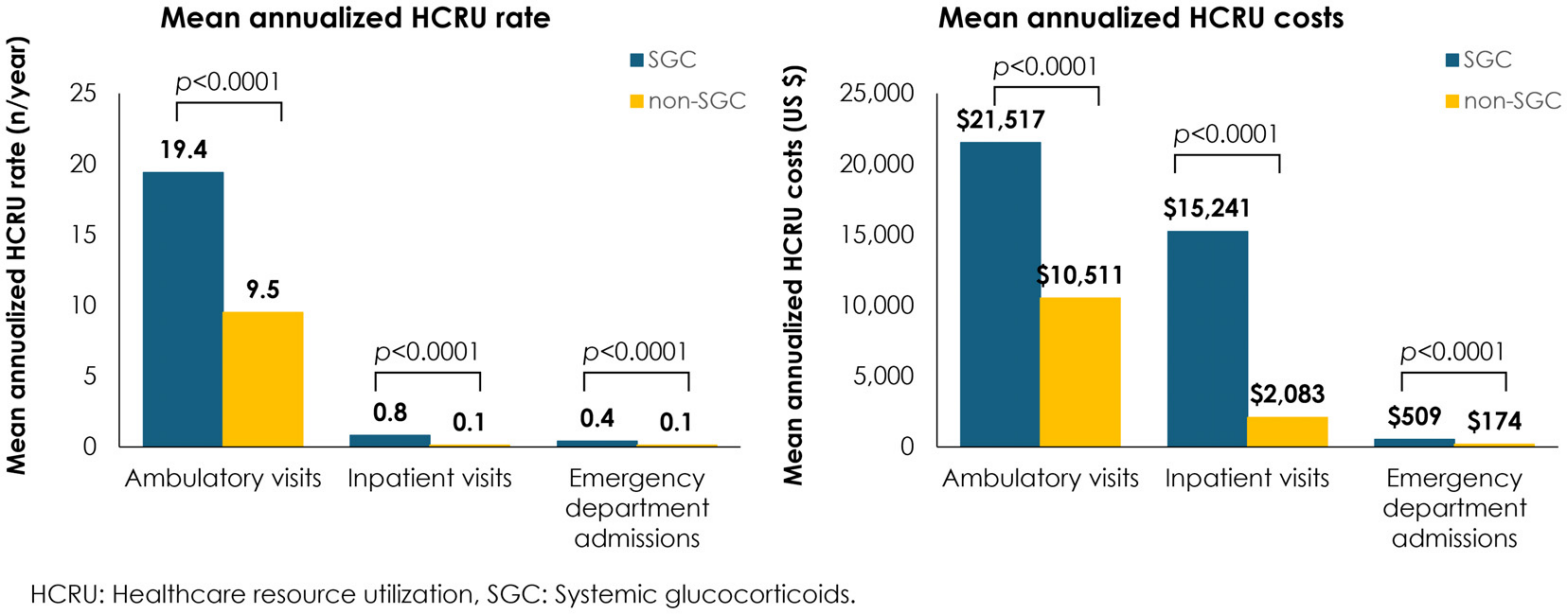
Mean annualized HCRU rate and cost by use/non-use of SGC.

Annualized mean HCRU rates and costs were significantly greater across all HCRU types for patients who received any of the four common SGCs vs those who did not receive SGC, including an eight-fold increase in inpatient visits, a four-fold increase in emergency department admissions, and twice as many ambulatory visits (**Figure 2**). Overall, the annual HCRU cost, including ambulatory visits, emergency department admissions, and inpatient visits was 37,526 USD for patients with IgAN who received any of the four common SGCs vs 12,957 USD for patients who did not receive SGCs.

### Time to kidney failure

**Figure 3:**
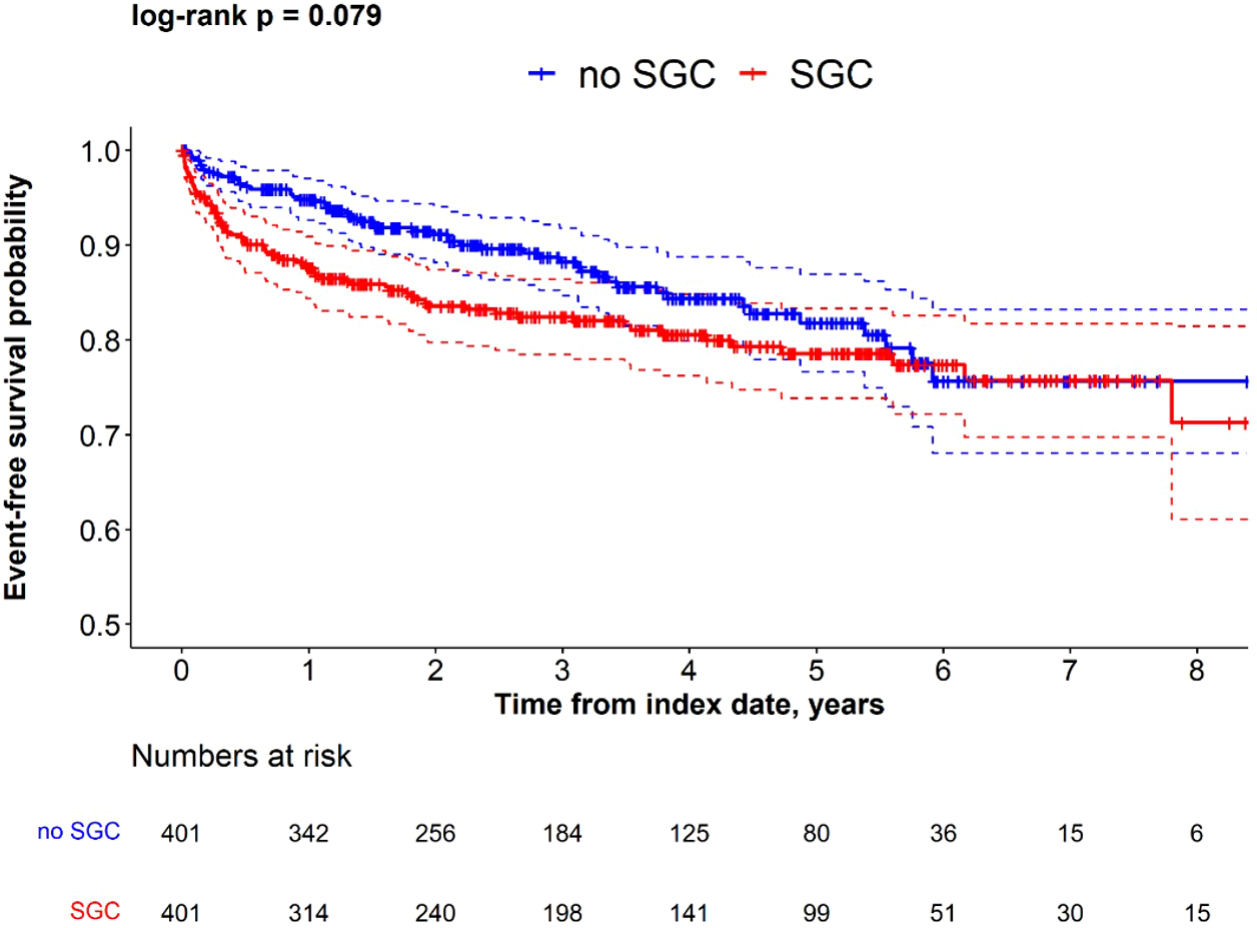
Kaplan-Meier survival for time to kidney failure in SGC and non-SGC cohorts among propensity score matched IgAN patients.

The proportion of patients who had kidney failure during the follow-up period was higher in patients who received any of the four commonly used SGCs compared with the non-SGC cohort (18% vs 13%, p=0.05), which was also reflected in the greater incidence rate (5.74 per 100 person-years in SGC cohort vs. 4.37 per 100 person-years in non-SGC cohort). The Kaplan-Meier analysis suggested a shorter time to kidney failure in IgAN patients in the SGC cohort vs. non-SGC cohort (log-rank p=0.079), especially in the first year after index date (**Figure 3**).

Overall, incidence of kidney failure was greater in the SGC cohort compared to the non-SGC cohort. The multivariable model, which adjusted for unbalanced characteristics at index date, confirmed the results of the unadjusted models, suggesting that the difference in kidney failure incidence between cohorts was independent from the differences of observed comorbidities at index/pseudo-index date. The Fine and Gray models indicated that the difference between cohorts was greater after 1 year from index (aHR at 1 year: 2.33, p=0.002), and progressively decreased at later time points (aHR at 2 years: 1.83, p=0.005; aHR at 3 years: 1.56, p=0.028; aHR at 5 years: 1.34, p=0.118) (**Table 3**). This suggests that the difference in kidney failure incidence is mainly restricted to the initial period after index date.

**Table 3:** Fine and Gray model for comparison of time to kidney failure in SGC cohort and non-SGC cohort at 1-5 years after index date.

	SGC cohort N=410	Non-SGC cohort N=401	Unadjusted model (M0)		Adjusted model (M1)	
Time after index date	Patients with kidney failure		HR (95% CI)	p-value	HR (95% CI)	p-value
1 year	48	20	2.501 (1.488;4.206)	**0.0005**	2.325 (1.382; 3.914)	**0.0015**
2 years	61	32	1.999 (1.306; 3.060)	**0.0014**	1.837 (1.198; 2.816)	**0.0053**
3 years	64	39	1.711 (1.151; 2.544)	**0.008**	1.562 (1.049; 2.326)	**0.028**
4 years	68	46	1.523 (1.048; 2.213)	**0.0273**	1.378 (0.948; 2.004)	0.0826
5 years	71	49	1.481 (1.030; 2.131)	**0.0343**	1.335 (0.929; 1.919)	0.1183

Significant P values indicated in bold. M0: unadjusted model. M1: model adjusted on the most significant variables retained after stepwise selection: hypertension, moderate or severe renal disease.

CI, confidence interval; HR, hazard ratio.

### Sensitivity analysis

The sensitivity analyses included only those IgAN patients identified using the ICD-10 code N02.8 (SGC cohort, n=367; non-SGC cohort, n=366), who comprised 91% of the main cohorts. The results from the main analyses regarding higher rates of AEs (**Supplementary Table S2**), kidney failure (**Supplementary Table S3**), HCRU (**Supplementary Table S4**), and costs (**Supplementary Table S5**) for the SGC cohort were overall confirmed in this subset.

## Discussion

This real-world retrospective, observational study investigated the incidence of AEs, HCRU and costs, and rates of kidney failure in US patients with IgAN from a large EMR database of patients treated with dexamethasone, prednisone, prednisolone, or methylprednisolone (SGC) compared with patients never treated with SGCs. Compared with 401 matched patients who did not receive SGC therapy, 401 patients who used SGC had:

- a significantly greater incidence of severe infections requiring hospitalizations and other AEs [including hypertension, arthralgia, dyspnea, fatigue, upper respiratory tract infection (URTI), peripheral/face edema, increased white blood cell (WBC) count, dyspepsia, weight gain],
- higher annual costs and HCRU rates for ambulatory visits, hospitalizations, and ER admissions, and
- higher rates of kidney failure, after adjustment for potential confounders at index date.

STOP-IgAN, a recently conducted, large, randomized, controlled trial investigated whether additional immunosuppression (cyclophosphamide, azathioprine, prednisolone) added to standardized supportive care could provide renal benefits in patients with progressive IgAN (n=162). During a three-year follow-up, the addition of immunosuppressive therapy to optimized supportive care led to significant reduction in proteinuria, but without providing substantial benefits in kidney preservation or prevention of eGFR decline. Moreover, patients treated with SGC experienced more severe infections, impaired glucose tolerance, and weight increase than patients in the supportive care group, especially during the first year of treatment (9). The TESTING trial, published after the KDIGO 2021 guidelines, evaluated the long-term efficacy and safety of oral methylprednisolone compared with placebo among 262 participants receiving routine RAS inhibitor therapy. Patients who had oral methylprednisolone had a significantly reduced risk of the composite outcome of kidney function decline, kidney failure, or death due to kidney disease (hazard ratio, 0.53). However, similar to our study results, the risk of serious AEs, most notably serious infections, was greater (10.9% vs 2.8%) in patients receiving glucocorticoids (8).

Based on available evidence, the KDIGO 2021 guidelines indicated the lack of an established clinical benefit in SGC treatment for IgAN, recommending that SGC treatment should be carefully considered in patients with IgAN and avoided in those patients with eGFR <30 mL/min/min^2^ as well as in patients with latent infections and with comorbidities (i.e., diabetes, obesity, secondary disease such as cirrhosis, active peptic ulceration, dementia, and severe osteoporosis) (1).

Overall, our findings are in line with the results of the STOP-IgAN and TESTING trials and support the latest KDIGO recommendations by showing that patients with IgAN treated with four commonly used SGCs had greater incidence of severe infections and AEs as compared to patients who were not treated with SGCs. In our study, the overall annual healthcare costs in patients with IgAN were higher in those treated with SGCs than in those who did not receive SGC therapy (37,500 USD vs 13,000 USD). Although no other studies compared the healthcare costs in IgAN patients by treatment received (7), a recent real-world study on IgAN patients in the Optum’s Market Clarity database reported the annual healthcare costs in these patients, which ranged between 17,500 USD and 44,800 USD depending on their proteinuria level (<1 g/day vs ≥1 g/day, respectively) (5). Overall, our estimates for IgAN management costs are in line with the findings of this recent study.

A strength of this study is the use of longitudinal data from a large, nationally representative standardized EMR database, which included a sufficiently robust sample size to reflect real-world clinical outcomes and HCRU in a representative group of patients with IgAN from the general population for this rare disease.

However, some limitations for this study should be noted. First, the identification of patients with IgAN is based on physician-recorded diagnosis code using a limited set of ICD-10, without additional pathological information from kidney biopsy. As no specific ICD-10 codes were available for IgAN at the time this study was implemented, some misclassification on diagnosis might have occurred. Despite IgAN being a well-recognized condition, a specific ICD-10 code for IgAN was introduced after this dataset was curated (N02.B, effective on October 2023) (17); until this date, IgAN cases were commonly coded as N02.8 (“recurrent and persistent hematuria with other morphologic changes”) as described in the Methods section. As patients identified with the code N04.1 might identify patients with other and more severe conditions than IgAN, sensitivity analyses using only patients identified with the code N02.8 were conducted, which yielded consistent results to the main analysis (**Supplementary Tables S2**-**S5**). Second, due to the nature of the real-world EMR database, standard of care clinical practice might impact the availability of data at timepoints of interest, and some patient groups might have more data available than others (e.g., patients with more severe symptoms may have more frequent clinic visits while patients with milder disease might have less follow-up data). However, as the number of patients in each cohort was similar at each timepoint in terms of available or missing key data (e.g., eGFR measurements), this might have affected the comparative analyses only to a minor extent. Third, there could be residual confounding due to unbalanced characteristics affecting IgAN disease severity. To minimize these differences, PS matching and multivariable models were used to balance patients’ characteristics at baseline and to adjust for unbalanced variables at index date at index/pseudo-index date between SGC and non-SGC cohorts. Residual confounding on unmeasured variables, however, cannot be completely ruled out. Fourth, the cause of HCRU is not reported in the database. Although the groups were PS-matched for several comorbidities and conditions at baseline, we cannot definitively determine whether the observed increase in HCRU in the SGC-treated group is attributable to SGC-associated AEs, unbalanced underlying disease severity, or other unmeasured factors. Fifth, there is a relevant proportion of patients with missing data for important variables, such as proteinuria, eGFR, or CKD stage at baseline. Although CKD stage could not be assessed for nearly one-third of patients per group, the distribution of missing values was balanced across exposure groups in the PSM. If the characteristics of patients with missing CKD data are similar across exposure groups, the presence of missing values likely increased random error, leading to greater variance in estimates, wider confidence intervals, and reduced power to detect significant differences. Sixth, although race/ethnicity was recorded in the study and balanced across exposure groups, other social determinants such as education, occupation, and income that can influence HCRU and AE reporting are not reported in the dataset. Groups with limited healthcare access may have underreported AEs due to fewer healthcare visits, potentially biasing comparisons. Seventh, patients were identified based solely on ICD-10 codes. As a result, some patients diagnosed before October 2015 (when ICD-10 had mandatory implementation in the US) who did not have a subsequent diagnosis after October 2015 may have been excluded, and other patients may have an earlier index date. This selection was not differential between the SGC and non-SGC groups, making it unlikely to impact the conclusions. Lastly, lack of information on SGC dosing and duration of treatment precluded our ability to isolate specific SGC treatments and doses for further analysis, and information on newer treatments for IgAN such as sparsentan and targeted release formulation-budesonide (Nefecon) (18, 19), were not assessed in this study due to the small number of patients (<5) in the TriNetX Dataworks database at the time of the analysis.

## Conclusions

Our study demonstrated that treatment of IgAN patients with the four commonly used SGC was associated with significant increases in adverse reactions and healthcare resource use rates and costs when compared with patients who did not receive SGC. We also found that receipt of SGC did not protect patients from kidney failure – neither in terms of rate of kidney failure nor for time to kidney failure. This real-world analysis underscores the importance of carefully assessing benefits and risks before treating IgAN patients with SGC.

## Data Availability

The data analyzed in this study are provided by TriNetX. Requests to access these datasets should be directed to trinetX.com.

## Supplementary material

Supplementary Table 1 Distribution of baseline characteristics at IgAN diagnosis in SGC cohort and non-SGC cohorts before and after propensity score matching. PS, propensity score; SGC, systemic glucocorticoids; IgAN, immunoglobulin A nephropathy; CKD, chronic kidney disease; CCI, CharlSson Comorbidity Index ^in the 12 months prior of the index date.

Supplementary Table 2 AE and AE of special interest stratified by use/non-use of SGC among IgAN patients identified using the N02.8 diagnosis code.

Supplementary Table 3 Fine and Gray model results for time to kidney failure in SGC cohort and non-SGC cohort identified using N02.8 diagnosis code only 1-5 years after index date. Significant P values indicated in bold. M0: unadjusted model. M1: model adjusted on the most significant variables retained after stepwise selection: hypertension, moderate or severe renal disease. HR, hazard ratio; CI, confidence interval.

Supplementary Table 4 Sensitivity analysis: mean annualized healthcare resource utilization rates by use of systemic glucocorticoids among propensity score matched IgAN patients identified using the diagnosis code N02.8. Rates are expressed as mean [standard deviation] per patient per year.

Supplementary Table 5 Sensitivity analysis: mean annualized healthcare costs (in USD) by use of systemic glucocorticoids among propensity score matched IgAN patients identified using the diagnosis code N02.8. Costs are expressed as mean [standard deviation] per patient per year.

## Appendix: List of Codes

**Table A1:** ICD-10 code for comorbidities of interest and Charlson Comorbidity Index.

Variable	ICD-10 code
Comorbidities of interest	
Asthma	J45*
Eczema	L20*
Anaphylactic Allergies	T78.0*,T78.2*
Chronic Obstructive Pulmonary Disease	I27.8*,I27.9*,J40*,J41*,J42*,J43*,J44*,J45*,J46*,J47*,J60*,J61*,J62*,J63*,J64*,J65*,J66*,J67*,J68.4*,J70.1*,J70.3*
Inflammatory Bowel Disease	K50*, K51*
Rheumatic Diseases (lupus)	M32*
Multiple Sclerosis	G35*
Charlson comorbidity components	
Myocardial Infarction	I21*,I22*,I25.2*
Congestive Heart Failure	I09.9*,I11.0*,I13.0*,I13.2*,I25.5*,I42.0*,I42.5*,I42.6*,I42.7*,I42.8*,I42.9*,I43*,I50*,P29.0*
Peripheral Vascular Disease	I70*,I71*,I73.1*,I73.8*,I73.9*,I77.1*,I79.0*,I79.2*,K55.1*,K55.8*,K55.9*,Z95.8*,Z95.9*
Cerebrovascular Disease	G45*,G46*,I60*,I61*,I62*,I63*,I64*,I65*,I66*,I67*,I68*,I69*,H34.0*
Dementia	F00*,F01*,F02*,F03*,F051*,G311*,G30*
Chronic Pulmonary Disease	I27.8*,I27.9*,J40*,J41*,J42*,J43*,J44*,J45*,J46*,J47*,J60*,J61*,J62*,J63*,J64*,J65*,J66*,J67*,J68.4*,J70.1*,J70.3*
Connective Tissue Disease	M05*,M06*,M31.5*,M32*,M33*,M34*,M35.1*,M35.3*,M36.0*
Ulcer Disease	K25*,K26*,K27*,K28*
Mild Liver Disease	B18*,K70.0*,K70.1*,K70.2*,K70.3*,K70.9*,K71.3*,K71.4*,K71.5*,K71.7*,K73*,K74*,K76.0*,K76.2*,K76.3*,K76.4*,K76.8*,K76.9*,Z94.4*
Diabetes Without Complications	E10.0*,E10.1*,E10.6*,E10.8*,E10.9*,E11.0*,E11.1*,E11.6*,E11.8*,E11.9*,E12.0*,E12.1*,E12.6*,E12.8*,E12.9*,E13.0*,E13.1*,E13.6*,E13.8*,E13.9*,E14.0*,E14.1*,E14.6*,E14.8*,E14.9*
Paraplegia and Hemiplegia	G04.1*,G11.4*,G80.1*,G80.2*,G81*,G82*,G83.0*,G83.1*,G83.2*,G83.3*,G83.4*,G83.9*
Moderate or Several Renal Diseases	I12.0*,I13.1*,N03.2*,N03.3*,N03.4*,N03.5*,N03.6*,N03.7*,N05.2*,N05.3*,N05.4*,N05.5*,N05.6*,N05.7*,N18*,N19*,N25.0*,Z49.0*,Z49.1*,Z49.2*,Z94.0*,Z99.2*
Diabetes with Complications	E10.2','E10.3','E10.4','E10.5','E10.7','E11.2','E11.3','E11.4','E11.5','E11.7','E12.2','E12.3','E12.4','E12.5','E12.7','E13.2','E13.3','E13.4','E13.5','E13.7','E14.2','E14.3','E14.4','E14.5','E14.7'
Cancer	C00*,C01*,C02*,C03*,C04*,C05*,C06*,C07*,C08*,C09*,C10*,C11*,C12*,C13*,C14*,C15*,C16*,C17*,C18*,C19*,C20*,C21*,C22*,C23*,C24*,C25*,C26*,C30*,C31*,C32*,C33*,C34*,C37*,C38*,C39*,C40*,C41*,C43*,C45*,C46*,C47*,C48*,C49*,C50*,C51*,C52*,C53*,C54*,C55*,C56*,C57*,C58*,C60*,C61*,C62*,C63*,C64*,C65*,C66*,C67*, C68*,C69*,C70*,C71*,C72*,C73*,C74*,C75*,C76*,C81*,C82*,C83*,C84*,C85*,C88*,C90*,C91*,C92*,C93*,C94*,C95*,C96*,C97*
Moderate or Severe Liver Disease	I85.0*,I85.9*,I86.4*,I98.2*,K70.4*,K71.1*,K72.1*,K72.9*,K76.5*,K76.6*,K76.7*
Metastatic Solid Tumor	C77*,C78*,C79*,C80*
AIDS	B20*,B21*,B22*,B24*

**Table A2:** ICD-10 code for adverse events of special interest.

Variable	ICD-10 code
Peripheral Edema	R60.0
Hypertension	I10*, I11*, I12*, I13*, I15*, I16*
Muscle Spasms	M62.83*
Acne	L70*
Upper Respiratory Tract Infections	J00*, J01*, J02*, J03*, J04*, J05*, J06*
Face Edema	R60.0
Weight Increased	R63.5
Dyspepsia	R10.13, K30*
Arthralgia	M25.5*
Increase in White Blood Cell Counts	D72.829
Dermatitis	L20*, L21*, L27*, L30*
Fatigue	R53*
Dyspnea	R06.0*
Hirsutism	L68.0*
Severe Infection Requiring Hospitalization	A40.*, A41.*, A02.1, A26.7, A32.7, A42.7, A54.86, B37.7, R65.20, R65.21, T81.44X
New Onset of Diabetes Mellitus (with or without complications)	E10.0*,E10.1*,E10.6*,E10.8*,E10.9*,E11.0*,E11.1*,E11.6*,E11.8*,E11.9*,E12.0*,E12.1*,E12.6*,E12.8*,E12.9*,E13.0*,E13.1*,E13.6*,E13.8*,E13.9*,E14.0*,E14.1*,E14.6*,E14.8*,E14.9*,E10.2*,E10.3*,E10.4*,E10.5*,E10.7*,E11.2*,E11.3*,E11.4*,E11.5*,E11.7*,E12.2*,E12.3*,E12.4*,E12.5*,E12.7*,E13.2*,E13.3*,E13.4*,E13.5*,E13.7*,E14.2*,E14.3*,E14.4*,E14.5*,E14.7*,'E10.2','E10.3','E10.4','E10.5','E10.7','E14.2','E14.3','E14.4','E14.5','E14.7'
Confirmed Fracture	S02*, S12*, S22*, S32*, S42*, S52*, S62*, S72*, S82*, S92*
New Osteonecrosis	M87*
Gastrointestinal Bleeding that Required Hospitalization	K92.2
Reported Occurrence of Cataract Formation	H26*, H28*
Reported Onset of Glaucoma	H40*, H42*
