# Supplementary material for: Real-world challenges associated with the use of four common systemic glucocorticoids in a United States IgAN cohort
Source: Front Nephrol. 2025 Apr 24;5:1574239. doi: 10.3389/fneph.2025.1574239 (PMC12058894; doi:10.3389/fneph.2025.1574239)
Supplement: Supplementary Table 1 — Distribution of baseline characteristics at IgAN diagnosis in SGC cohort and non-SGC cohorts before and after propensity score matching. PS, propensity score; SGC, systemic glucocorticoids; IgAN, immunoglobulin A nephropathy; CKD, chronic kidney disease; CCI, CharlSson Comorbidity Index ^in the 12 months prior of the index date. [file DataSheet1.pdf]

Supplementary Material

Table S1. Distribution of baseline characteristics at IgAN diagnosis in SGC cohort and non-SGC cohorts before and after propensity score matching

	**before PS matching**				**post PS matching**			
	SCS (n= 554)	Non-SCS (n= 620)	p-value	standardized difference %	SCS (n= 401)	Non-SCS (n= 401)	p-value	standardized difference %
**Age at IgAN diagnosis, years**			0.41	3.53			0.28	6.45
Mean (SD)	41.49 [18.18]	40.84 [18.27]			41.15 [18.41]	39.98 [17.77]		
Median (IQR)	42 [29; 56]	41 [27; 54]			42 [29; 56]	40 [27; 53]		
**Age at diagnosis, years, n (%)**			1				0.56	
< 18	73 (13.22%)	82 (13.25%)		-0.14	56 (13.97%)	55 (13.75%)		0.72
18-<65	419 (75.91%)	470 (75.93%)		-0.41	301 (75.06%)	310 (77.5%)		-5.27
≥ 65	60 (10.87%)	67 (10.82%)		0.08	44 (10.97%)	35 (8.75%)		7.54
missing	2	1		3.91	0	1		-7.07
**Sex, n (%)**			0.73	-2.05			0.94	0.5
Male	291 (52.53%)	332 (53.55%)			220 (54.86%)	219 (54.61%)		
Female	263 (47.47%)	288 (46.45%)			181 (45.14%)	182 (45.39%)		
**Patient Regional Location, n(%)**			0.35				0.30	
Midwest	122 (22.02%)	112 (18.06%)		9.9	90 (22.44%)	70 (17.46%)		9.9
Northeast	198 (35.74%)	222 (35.81%)		-0.14	144 (35.91%)	142 (35.41%)		-0.14
South	156 (28.16%)	197 (31.77%)		-7.9	110 (27.43%)	127 (31.67%)		-7.9
West	78 (14.08%)	88 (14.19%)		-0.33	57 (14.21%)	61 (15.21%)		-0.33
Unknown	0 (0.%)	1 (0.16%)		-5.68	0 (0.%)	1 (0.25%)		-5.68
**Race, n (%)**			0.59				0.69	
American Indian or Alaska Native	3 (0.54%)	3 (0.48%)		0.81	2 (0.5%)	1 (0.25%)		4.09
Asian	58 (10.47%)	61 (9.84%)		2.09	50 (12.47%)	42 (10.47%)		6.26
Black or African American	49 (8.84%)	48 (7.74%)		4	34 (8.48%)	31 (7.73%)		2.74
White	345 (62.27%)	414 (66.77%)		-9.42	242 (60.35%)	261 (65.09%)		-9.81
Unknown/Unspecified	99 (17.87%)	94 (15.16%)		7.3	73 (18.2%)	66 (16.46%)		4.61
**Hispanic/Latino, n (%)**	3 (0.54%)	3 (0.48%)	0.61	1.49	58 (14.46%)	55 (13.72%)	0.95	2.15
**IgAN type, n (%)**			0.44	4.49			0.90	0.89

N028	505 (91.16%)	557 (89.84%)			367 (91.52%)	366 (91.27%)		
N041	49 (8.84%)	63 (10.16%)			34 (8.48%)	35 (8.73%)		
**CKD stage, n (%) prior index date**			0.004				0.67	
Stage 1 or stage 2	147 (26.53%)	192 (30.97%)		-9.81	123 (30.67%)	120 (29.93%)		1.63
Stage 3	147 (26.53%)	110 (17.74%)		21.3	80 (19.95%)	90 (22.44%)		-6.1
Stage 4	44 (7.94%)	57 (9.19%)		-4.47	30 (7.48%)	23 (5.74%)		7.03
missing	216 (38.99%)	261 (42.1%)		-6.33	168 (41.9%)	168 (41.9%)		0
**Index year, n (%)**			0.45				1	
2011-2016	187 (33.75%)	195 (31.45%)		4.91	132 (32.92%)	132 (32.92%)		0
2017-2018	169 (30.51%)	181 (29.19%)		2.87	123 (30.67%)	123 (30.67%)		0
2019-2020	120 (21.66%)	159 (25.65%)		-9.39	89 (22.19%)	89 (22.19%)		0
2021-2022	78 (14.08%)	85 (13.71%)		1.07	57 (14.21%)	57 (14.21%)		0
**Hypertension, n (%)**	319 (57.58%)	326 (52.58%)	0.09	10.07	221 (55.11%)	215 (53.62%)	0.67	3
**CCI**			0.41	5.31			0.83	2.32
Mean (SD)	1.63 [2.01]	1.53 [1.86]			1.58 [1.99]	1.54 [1.87]		
**CCI, n (%)**			0.15				0.97	
0	223 (40.25%)	267 (43.06%)		-5.71	171 (42.64%)	173 (43.14%)		-1.01
1	57 (10.29%)	46 (7.42%)		10.11	34 (8.48%)	33 (8.23%)		0.9
2	163 (29.42%)	200 (32.26%)		-6.14	117 (29.18%)	121 (30.17%)		-2.18
≥3	111 (20.04%)	107 (17.26%)		7.14	79 (19.7%)	74 (18.45%)		3.17
**RAS inhibitor therapy ^**	178 (32.13%)	189 (30.48%)	0.54	3.55	126 (31.42%)	121 (30.17%)	0.70	2.7

PS=propensity score; SGC=systemic glucocorticoids; IgAN=immunoglobulin A nephropathy; CKD=chronic kidney disease; CCI=Charlson Comorbidity Index ^in the 12 months prior of the index date

Table S2: AE and AE of special interest stratified by use/non-use of SGC among IgAN patients identified using the N02.8 diagnosis code

	SGC	non-SGC	
**Adverse Event (AE), n (%)**	**N=367**	**N=366**	**p-value**
Acne	19 (5.18%)	7 (1.91%)	**0.02**
Arthralgia	125 (34.06%)	75 (20.49%)	**<.0001**
Dermatitis	34 (9.26%)	21 (5.74%)	0.07
Dyspepsia	30 (8.17%)	13 (3.55%)	**0.008**
Dyspnea	98 (26.7%)	42 (11.48%)	**<.0001**
Fatigue	91 (24.8%)	54 (14.75%)	**0.0006**
Hypertension	266 (72.48%)	225 (61.48%)	**0.002**
(incident hypertension)*	59 (41.26%)	48 (27.59%)	**0.01**
Increase in WBC count	31 (8.45%)	11 (3.01%)	**0.002**
Peripheral/face edema	51 (13.9%)	12 (3.28%)	**<.0001**
URTI	83 (22.62%)	47 (12.84%)	**0.0005**
Weight increased	14 (3.81%)	4 (1.09%)	**0.02**
**Adverse Event of Special Interest (AESI), n (%)**			
Confirmed fracture	17 (4.63%)	21 (5.74%)	0.5
Gastrointestinal bleeding that required hospitalization	8 (2.18%)	2 (0.55%)	0.11
New onset of diabetes mellitus	60 (16.35%)	49 (13.39%)	0.26
Reported onset of glaucoma	8 (2.18%)	9 (2.46%)	0.8
Severe infection requiring hospitalization	10 (2.72%)	1 (0.27%)	**0.006**

*Among patients without hypertension diagnosis before index date. AE, adverse event; AESI: adverse event of special interest; GI, gastrointestinal; SGC, systemic glucocorticoids; URTI, upper respiratory tract infections; WBC, white blood cell.

Table S3. Fine and Gray model results for time to kidney failure in SGC cohort and non-SGC cohort identified using N02.8 diagnosis code only 1-5 years after index date

	SGC cohort	Non-SGC cohort	Unadjusted model (M0)		Adjusted model (M1)	
N	367	366				
Time after index date	Patients with kidney failure		HR (95% CI)	p-value	HR (95% CI)	p-value
1 year	44	20	2.301 (1.359; 3.895)	**0.0019**	2.082 (1.228; 3.530)	**0.0065**
2 years	53	30	1.852 (1.186; 2.892)	**0.0067**	1.639 (1.047; 2.565)	**0.0307**
3 years	54	33	1.708 (1.109; 2.630)	**0.015**	1.507 (0.977; 2.326)	0.0637
4 years	58	38	1.568 (1.042; 2.360)	**0.031**	1.371 (0.910; 2.067)	0.131
5 years	61	41	1.511 (1.017; 2.246)	**0.041**	1.314 (0.885; 1.952)	0.175

Significant P values indicated in **bold**. M0: unadjusted model. M1: model adjusted on the most significant variables retained after stepwise selection: hypertension, moderate or severe renal disease. HR: hazard ratio; CI: confidence interval.

Table S4. Sensitivity analysis: mean annualized healthcare resource utilization rates by use of systemic glucocorticoids among propensity score matched IgAN patients identified using the diagnosis code N02.8

	**SGC**	**Non-SGC**	**p-value**
	N=367	N=366	
Ambulatory visits	19.04 [23.7]	9.48 [17.73]	**<.0001**
Inpatient visits	0.78 [2.99]	0.11 [0.51]	**<.0001**
ER admissions	0.42 [1.11]	0.15 [0.6]	**<.0001**

Rates are expressed as mean [standard deviation] per patient per year.

Table S5. Sensitivity analysis: mean annualized healthcare costs (in USD) by use of systemic glucocorticoids among propensity score matched IgAN patients identified using the diagnosis code N02.8

	SGC	Non-SGC	p-value
	N=367	N=366	
Ambulatory visits	21,132 [26,309]	10,524 [19,682]	**<.0001**
Inpatient visits	15,378 [65632]	2,173 [17824]	**<.0001**
ER admissions	510 [1,347]	181 [722]	**<.0001**

Costs are expressed as mean [standard deviation] per patient per year.
